# Spreading depolarization drives distant connectivity changes in the focally injured cortex

**DOI:** 10.1371/journal.pone.0362090

**Published:** 2026-11-03

**Authors:** Daria A. Lachinova, Maria P. Smirnova, Ilya V. Sysoev, Lyudmila V. Vinogradova

**Affiliations:** 1 Institute of Higher Nervous Activity and Neurophysiology, Russian Academy of Sciences, Moscow, Russia; 2 Saratov State University, Saratov, Russia; 3 Peter the Great St. Petersburg Polytechnic University, St. Petersburg, Russia; University of Modena and Reggio Emilia, ITALY

## Abstract

Cortical spreading depolarization (SD), a slow-propagating wave of transient cellular depolarization, reliably occurs after acute brain injury but usually remains undetected. Focal brain injury is known to produce a network-level dysfunction that changes the strength and direction of connectivity far beyond lesion site (connectional diaschisis) that can underlie post-injury neurological dysfunction. We suggested that injury-induced SD could contribute to connectional diaschisis and studied the effects of SD on functional and effective connectivity in neural networks supporting cognitive and sensory functions. Electrographic recordings of cortical activity were obtained in freely behaving rats immediately after focal micro-injury of the frontal cortex. The injury-induced SD propagated over the cortex of the ipsilesional hemisphere but never invaded the contralesional cortex. Strength and direction of connectivity were estimated using non-directional (mutual information function) and directional (transfer entropy) measures calculated for recordings from the retrosplenial (a key hub of the default-mode network) and somatosensory cortex. Mutual information analysis showed initial hypercoupling within the injured hemisphere (during SD) followed by longer interhemispheric decoupling (after SD termination). During post-SD hypoconnectivity, influence of the uninjured cortex on the injured one increased in both cortical regions. The retrosplenial cortex, component of the default-mode network, showed strong interhemispheric coupling during the baseline period and complex changes in connectivity after SD. Therefore, SD arising in the injured cortex disturbs long-range connectivity in distant cortical regions and causes widespread network alterations involving the default-mode network. Given the key role of the network in cognition, connectivity changes produced by injury-induced SD could contribute to the mechanisms of cognitive deficit after brain injury.

## Introduction

Cortical spreading depolarization (SD) is a wave of intense cellular depolarization propagating over the cortex with low (3–5 mm/min) velocity and causing transient changes in neuronal activity, metabolism and blood flow in the affected tissue [1–3]. Clinical studies have shown that SD is a reliable component of the cortical response to acute brain injury but conventional scalp EEG is unable to detect SD. Therefore, in most clinical situations the occurrence of SD remains unrecognized. In patients with stroke or brain trauma, intracranial recordings detected clusters of recurrent cortical SDs during acute and subacute periods [4,5]. Because the clusters can contribute to secondary brain injury, cortical SD is currently recognized as an important target for monitoring and therapy in patients with acute brain insults [5].

Focal brain injury is known to produce functional changes far beyond the lesion site (diaschisis). The distant changes occur within minutes after acute insults and gradually regress over time [6,7]. Connectional diaschisis, a network-level dysfunction observed after stroke or traumatic brain injury, includes changes in patterns, strength and direction of connectivity in intact areas far from injury [6]. Functional imaging studies have shown that a local cortical damage produces widespread changes in connectivity in both hemispheres and between them [8]. In patients with brain injury, connectivity changes in the motor cortex are thought to contribute to motor impairment and normalization of connectivity correlates with recovery of motor function [8,9]. Now, connectivity analysis is suggested to provide new information on the pathophysiological mechanisms of acute brain injury, its consequences and therapy [6].

Being locally induced, cortical SD reliably invades distant regions of the ipsilateral hemisphere [1,2]. Therefore, SD waves that arise in the injured brain could contribute to diaschisis, including the connectional one. Previously, we have found that the propagation of SD to unimodal (motor or sensory) cortical regions transiently disrupts interhemispheric functional connectivity [10–12]. However, it is unknown whether SD affects connectivity in transmodal cortical networks, particularly in the default-mode network (DMN). The widely distributed network increases its activity at rest and plays a crucial role in internally-directed cognition. Abnormal patterns of DMN connectivity have been found in patients with traumatic brain injury, schizophrenia, autism, depression [13,14]. Resting-state functional connectivity in the DMN is extensively studied using a functional neuroimaging approach and DMN connectivity measures are suggested as a biomarker of cognitive state for clinical conditions [14,15]. Disconnectivity within the DMN network is supposed to contribute to cognitive deficit after acute brain insults [16].

We hypothesized that SD triggered by an acute focal injury of the cortex could contribute to connectional diaschisis and mediate distant connectivity changes in cortical networks that support cognitive and sensory functions. To examine the suggestion, we studied the effect of SD developed in response to a distant focal injury of the cortex on functional and effective connectivity in intact cortical regions, the retrosplenial and somatosensory cortices. The retrosplenial cortex (posterior cingulate cortex in humans) is a key hub of the default-mode network and transmodal association cortex that supports high cognitive functions [13,17]. The somatosensory cortex is a unimodal sensory cortex that plays a key role in processing sensory information. Changes in strength and direction of intracortical (interhemispheric and intrahemispheric) connections were estimated using non-directional (the mutual information function) and directional (transfer entropy) measures. Given that electrophysiological-based approach provides direct assessment of functional connectivity with high temporal resolution, we calculated the connectivity measures using electrographic recordings of cortical activity obtained in freely behaving animals immediately after remote micro-injury of the cortex.

## Materials and methods

### Animals

All experimental procedures were designed according to ARRIVE guidelines and the study was approved by the Ethics Committee of the Institute of Higher Nervous Activity and Neurophysiology (protocol No. 1, 11.02.2022). Wistar rats (3–4 months, males, 285–400) were received from Stolbovaya Animal Breeding Center (Moscow, Russia). Rats were housed in the animal facility in individual cages under controlled environmental conditions (a 12-h light-dark cycle, lights on at 7:00 A. M., 20–23°C) with free access to food and water. Well-trained and experienced stuff cared for animals and monitored their behavior and health every day. Efforts were made to minimize the number of used animals and their suffering. Rats were allowed to habituate for 2 weeks prior to electrode implantation surgery.

### Surgery

Guide cannula for producing focal cortical micro-injury and electrodes for recording local field potentials (LFP) were stereotactically implanted under isoflurane anesthesia two weeks before the onset of experiments. Stainless-steel guide cannulas (outer diameter 0.6 mm; inner diameter 0.4 mm) aimed at middle layers of the frontal cortex (AP ±1.2; ML ±2.3) of the left and right hemispheres. The same length stylet was inserted into the guide cannula to prevent its obstruction. To record LFP, electrodes made of 0.25-mm-diameter teflon-coated silver wire (World Precision Instruments) were implanted in homotopic sites of the somatosensory (AP −2.8; ML ±4.8) and retrosplenial (AP −4.8; ML ±1.1) cortex of the two hemispheres. The reference electrode was placed over the cerebellum. Electrodes and guide cannulas were soldered to a pin connector and were fixed on the skull with acrylic cement. Experiment was performed 2 weeks after the surgery. During the post-surgery period, rats were injected with antibiotic amoxicillin (0.3 ml, i. m.) and glucose solution (1 ml, s. c.). All seven implanted rats were used in experiments; none died during the post-surgery period.

### Experimental design

Experiments were performed in awake freely behaving rats with simultaneous LFP recording and video-monitoring of behavior. Each rat was individually placed in a shielded experimental chamber (60×40×40 cm) and the implanted connector was attached to the recording cable. Electrical activity of the cortex (in a frequency band from 0 to 45 Hz) was recorded with a four-channel, high-input impedance (1gΩ) DC amplifier and A/D converter (E14-440, L-Card, Russia). The data were stored (1 kHz sampling rate, i. e. Δt=1 ms sampling interval) and analyzed off-line. After a 5-min period of habituation in the chamber and a 15-min recording baseline cortical activity, a microinjury of the frontal cortex of one hemisphere was performed and 15 minutes later identical injury of the symmetrical site in another hemisphere was produced. Microinjury was induced by a method described previously [18,19]. Briefly, the needle was introduced into cortical parenchyma via the implanted guide cannula and extended 1.0 mm from its tip. Animals were awake during the microinjury procedure and afterward, exhibiting locomotor or grooming behaviors. After the end of the experiment, rats were returned to their home cages; three days later all seven animals were euthanized using an overdose of anesthetics. None animal showed endpoint criteria for euthanasia (weight changes, abnormal behavior, neurological deficit etc.) or died before the end of the experiment.

Recordings of experimental signals of 400 s length from the somatosensory and retrosplenial cortices of both hemispheres were obtained before and after focal cortical injury of each hemisphere. Occurrence of SD was defined by characteristic high-amplitude negative potential shift. Out of 7 rats with implanted electrodes, one rat was excluded from analysis due to incorrect localization of guide cannulas. Six artefact-free LFP recordings were selected for the connectivity analysis (*L* = 6).

### Data preprocessing

In each channel, the experimental time series of local field potentials *v*(*t*) was characterized by a slow trend of the mean of *v*(*t*) and occasional artifacts. We removed the trend by extracting the mean (1) in the moving time window with the window semilength *T* = 1 s:


$$\begin{array}{cc} V_{n} & =\frac{1}{\left(2m+1\right)}\sum_{i=n-m}^{n+m}v_{i}, \end{array}$$ (1)

$$\begin{array}{cc} U_{n} & =v_{n}-V_{n}, \end{array}$$ (2)

where $v_{n}$ is a value measured in the time moment $t_{n}$, $V_{n}$ is a trend in this time moment (moving average) and $U_{n}$ is a corresponding detrended value, m=T/Δt. By means of this numerical procedure, the signal was divided into two components: the detrended (fast component) signal *U*(*t*) which was further analyzed and the trend *V*(*t*) (slow component) which included a negative DC potential shift associated with SD. Then, high amplitude artifacts were removed from *U*(*t*). Some of them were signal transitions beyond the ADC (analog *t*o digital converter) dynamical range, so it was only necessary *t*o add 10 or −10 mV to the signal. Recordings with more complex and long-lasting artifacts were excluded from the analysis completely or partly.

### Methods for connectivity reconstruction

Here, we applied two measures for functional connectivity estimation. The first one is a mutual information function *MI*, which is a very popular and relatively simple nonlinear measure of signal similarity described, e.g., by [20] and used in neuroscience for at least 40 years [21,22]. There are different estimators for calculation of *MI* from experimental data (time series). We used the estimator proposed by [23] and based on [24] entropy since it demands a few data, being successfully applied in neuroscience in the moving window when studying other disorders like epilepsy [25,26]. It has also been recently applied to estimation of intracortical connectivity dynamics associated with SD [10,11]. However, *MI* cannot reveal the connectivity direction and measures only signal similarity or synchrony. To reveal the connectivity direction, we used another measure from information theory — transfer entropy *TE* [27]. The transfer entropy can also be considered as a version of nonlinear Granger causality [28] (the main measure of Granger causality is often referred as *prediction improvement* — *PI*), and there are direct analytical proofs that for some very simple signals there are explicit formulae to calculate *TE* from *PI* and vice verse, but for complex signals this is not the case, see [29] for instance.

Both measures were calculated in 20-s sequential nonoverlapping time epochs. For the control series the whole recording (from 800 to 1100 s) was used to calculate the baseline values of *TE* and *MI*. When analyzing the response to SD, 400 s of recording were used, obtained directly after wave initiation (*K* = 20 epochs). For each *i*-th (i=1,2,...,K) time epoch for each recording the values of *MI* and *TE* were calculated in four channel pairs, given four values of *MI* and eight values of *TE* (due to directionality for each channel pair there could be two values of *TE* and the only value of *MI* since this measure is indirectional). Next, *MI* and *TE* were calculated for two intrahemispheric channel pairs (one corresponding to the affected hemisphere and the other corresponding to nonaffected one) for the symmetric channels: in the somatosensory cortex: $MI_{s}$, $TE_{sa\to sn}$ and $TE_{sn\to sa}$ and in the retrosplenial cortex: $MI_{r}$, $TE_{ra\to rn}$ and $TE_{rn\to ra}$. Then, *MI* and *TE* were calculated for two intrahemispheric channel pairs: in the affected hemisphere: $MI_{a}$, $TE_{sa\to ra}$ and $TE_{ra\to sa}$ and in the nonaffected one: $MI_{n}$, $TE_{sn\to rn}$ and $TE_{rn\to sn}$. Here and further, the notation is as follows: *a* stays for the affected hemisphere and *n* — for the nonaffected one, *s* — for the somatosensory cortex and *r* — for the retrosplenial cortex.

### Mutual information function

Mutual information function *MI* (3) can be considered as a generalization of correlation function, which takes into account all types of signal inter-dependencies: not only linear as correlation does, but also nonlinear.


$$\begin{array}{c} MI(X,Y)=H(X,Y)-H(X|Y)-H(Y|X), \end{array}$$ (3)

where *H*(*X*,*Y*) is the joint entropy of signals *X* and *Y* and *H*(*X*|*Y*) and *H*(*Y*|*X*) are conditional entropies.

If the entropies were calculated by mean of estimation of probability density from bins (i.  e. by calculating the probabilities, $p_{Y}(j)$ and $p_{X,Y}(i,j)$ that the corresponding value $\left(x_{n},y_{n}\right)$ belongs to *i*-th bin by *X* and *j*-th bin by *Y*), the calculation formula would be (4).


$$\begin{array}{c} MI(X,Y)=\sum_{i,j}p(i,j)\log\frac{p(i,j)}{p(i)p(j)}. \end{array}$$ (4)

The formula (4) is nonparametric and includes all possible nonlinear interdependences, but its application is problematic due to bad statistical properties of bin-based estimates: large errors for significantly nonuniform distributions together with demanding too many data for accurate estimation. We have to say that the differential entropy still has troublesome properties regardless the way it is calculated [30]. Difficulties in calculating *MI* forced scientists to use different parametric nonlinear correlation coefficients instead, see the popular approach by [31] for instance. Such coefficients may work fine for some dependencies and poorly for others due to different actual nonlinearities, which are unknown in practice, but have to be explicitly approximated.

Actually, the problem of *MI* calculation from short series (or samplings) was mostly solved in [24] with introducing a new estimator based on nearest neighbors instead of direct use of bins, but this work had bin unknown in neuroscience until the calculation algorithm was reformulated in [23]. Tests and comparisons showed this approach to be the best among others both by precision and computation complexity, see [32]. Since the problem of *MI* estimation is now mostly solved, different nonlinear correlation coefficients are no more relevant and we prefer direct use the algorithm by [23].

To calculate *MI* following [24] and [23] one should introduce the distance between data points $\left(x_{i},y_{i}\right)$ and $\left(x_{j},y_{j}\right)$ as a maximum of absolute values (5) rather than by means of Euclidean norm:


$$\begin{array}{c} d_{i,j}=\max(|x_{i}-x_{j}|,|y_{i}-y_{j}|). \end{array}$$ (5)

Then, for each data point $\left(x_{i},y_{i}\right)$ the *k*-th nearest neighbor is detected using (4) and numbers of points which are ether *X*-neighbors of $\left(x_{i},y_{i}\right)$ (this number is denoted as $n_{X}(i)$) or *Y*-neighbors of $\left(x_{i},y_{i}\right)$ (this number is denoted as $n_{Y}(i)$) are calculated. Then, the specific approximation of Shannon’s entropy using the distance in terms of (5) proposed by [24] gives us the formula (6):


$$\begin{array}{c} MI=\psi (N)+\psi (k)-\langle \psi \left(n_{X}(i)+1\right)+\psi \left(n_{Y}(i)+1\right)\rangle_{i=1,...,N} \end{array}$$ (6)

where *N* is the number of data points and ψ is a digamma-function. Estimates of *MI* ignore all continuous linear signal transformations, including signal shift and scaling.

The only method parameter is *k*. Usually, *k* > 1 is recommended. We tested our results for stability using *k* = 1, *k* = 6 and *k* = 10 and found no significant difference. So, we used *k* = 1 for all cases for calculation simplify.

### Transfer entropy

Transfer entropy was proposed in [27] as an alternative realization of the same concept as the nonlinear Granger causality [33]. It has been actively used in neuroscience for last decades [34]. The difference between the Granger causality and the transfer entropy is in using conditional entropies instead of forecasting models. Let us explain *TE* for a simple case of scalar signals. First, let us decide that we are interested to know whether the future of the signal from the system *X* measured as time series $\{x_{i}{\}}_{i=1}^{N}$ is determined not only by its current value, but also by the current value of a signal of another system *Y* measured as time series $\{y_{i}{\}}_{i=1}^{N}$. So, let us denote the time interval between future time moment (for which we are interested in causality) and the current time moment as τΔt and let us denote $z_{i}=x_{i+\tau }$ for further simplicity. The conditional entropy *H*(*z*|*x*) describes the amount of information about the future of the signal $\{x_{i}{\}}_{i=1}^{N}$ which cannot be found in its current state. And the conditional entropy *H*(*z*|*x*,*y*) describes how much information we obtain measuring future values of $\{x_{i}{\}}_{i=1}^{N}$ in addition to what we have can get both from signal $\{x_{i}{\}}_{i=1}^{N}$ and signal $\{y_{i}{\}}_{i=1}^{N}$ at the current moment. If these two entropies: *H*(*z*|*x*,*y*) and *H*(*z*|*x*) are significantly different (the only possible way is *H*(*z*|*x*) > *H*(*z*|*x*,*y*)), then we may accept the hypothesis that *Y* drives *X* since it helps to decrease uncertainty in the future of *X*.


$$\begin{array}{c} TE_{Y\to X}=H(z|x)-H(z|x,y). \end{array}$$ (7)

For ergodic signals one can estimate the entropies by averaging over time instead of averaging over different series. For the calculations, we used the similar nearest neighbor approach as for *MI*, which was proposed by different groups multiple times, see [35–37]. The calculation formula is (8).


$$\begin{array}{c} TE_{Y\to X}=\psi (k)+\langle \psi \left(n_{X}(i)+1\right)-\psi \left(n_{X,Y}(i)+1\right)-\psi (n_{X,Z}(i)+1)\rangle_{i=1,...,N-\tau }, \end{array}$$ (8)

where $n_{X}(i)$ is the same as in the (6) and $n_{X,Y}(i)$ and $n_{X,Z}(i)$ are calculated as numbers of neighbors for both *X* and *Y* or both *X* and *Z* accordingly.

Calculation of *TE* by means of (8) has two parameters: *k* and τ. We tested our method for *k* = 1, *k* = 6 and *k* = 10 at τ=1 and found no significant difference. We also tried to use the different value of τ (τ=8 in particular) to use some main time scale in the signal as it was recommended in [38] and found the results mostly similar, with the absolute values of estimates for *TE* at τ=8 being noisy (large irregular differences between neighbor time fragments). This may be a result of too simple model: to predict farther, we need to use a number of previous time points to predict the next value rather than a single point, i. e. we need to use the dimension of the model larger than one. But in such a case, we need to estimate four- or higher-dimensional entropies that is mostly impossible from real data. Therefore, we report the results obtained for *k* = 1 and τ=1 further.

### Statistical evaluation of connectivity estimates

The values of *MI* and *TE* like values of any other measure may become larger or lower due to some random factors [39,40]. The distributions of the *MI* and *TE* estimates are unknown, with being asymmetric due to the fact that any entropy is not negative. We may only guess that these distributions are far from the normal one, so any parametric statistics like t-test is not applicable. Therefore, we switched to min-max criterion.

First, we calculated the average values for the baseline activity for each channel pair (for *TE* in both directions independently) by averaging over all time intervals for all recordings (276 in total). Large averaging of these values led to a very small std of the mean (< 0.008 for *MI* and < 0.005 for *TE*), so we ignored it. Also, this allowed us to compare the baseline values for different channel pairs using two-sided t-test for two samplings for the mean.

Second, we calculated min and max values among all recordings (*L* = 6) for each metrics for each time interval for each channel pair (for *TE* also in both directions). Then, we considered the zero hypothesis that these values had the same mean as the baseline. If so, the probability for a particular value to be larger (smaller) than the average is $\frac{1}{2}$ and for all *L* values these probabilities were independent. This led to the decision, that the probability to obtain all *L* values larger (or lower) than the mean baseline value is $(\frac{1}{2}{)}^{L}$. So, if the minimal value was larger than the baseline value, we had to refute the hypothesis with the mistake probability (usually known as *p*-value) $p_{6}={\left(\frac{1}{2}\right)}^{6}=\frac{1}{64}\approx 0.0156$. This level of significance is usually acceptable. But since we had tested for coupling in 20 epochs, we had to make a correction for multiple comparison. Applying the Bonferroni correction means multiplying the value by 20, given $p_{6,c}\approx 0.31$ which is completely not acceptable. Therefore, to provide more significant results, we generally followed the approach by [41], considering two subsequent intervals together. If at each of them the results were significant at the level *p*_6_, this meant that at least at one of them (we do not know at which actually) the results were significant at the level $p_{6}^{2}\approx 2.44\cdot 10^{-4}$. This meant also that due to worse temporal resolution we considered only 10 independent intervals instead of *K* = 20. So, even after the Bonferroni correction we still have the *p*-value $p_{6,c}^{2}\approx 2.44\cdot 10^{-3}<1/400$ which is completely acceptable. Data on temporal characteristics of SD (depolarization phase duration and latency) in cortical regions were presented as mean ± standard deviation and compared using Wilcoxon signed-rank test with the significance threshold set at *p* < 0.05.

To sum up, the statistical analysis was performed using next tests: (1) two-sided t-test for comparison of baseline connectivity levels in different cortical regions; (2) non-parametric min-max criterion with the Bonferroni correction for evaluation of connectivity changes induced by SD with intervals grouped by pairs; (3) Wilcoxon signed-rank test for comparison of SD duration and latency in different cortical regions.

## Results

Most focal injuries(75%, 9/12) of the frontal cortex triggered a single unilateral SD that was detected 56 ± 10 s after injury in the somatosensory region and 78 ± 11 s in the retrosplenial region of the cortex and lasted 52 ± 16 s and 42 ± 8 s, respectively. Injury-induced SD appeared significantly earlier in the somatosensory cortex than in the retrosplenial ones (*p* = 0.0431) but the duration of SD did not differ significantly between the regions (*p* = 0.273, Wilcoxon test). Fig 1B shows typical time series of local field potentials in the non-affected — $v_{sn}(t)$ and affected — $v_{sa}(t)$ hemispheres of the somatosensory cortex (1st subplot), as well as in the retrosplenial cortex: non-affected — $v_{rn}(t)$ and affected — $v_{ra}(t)$ (3rd subplot). We see that the fast components of non-affected $U_{sn}(t)$ and affected $U_{sa}(t)$ hemispheres from somatosensory cortex and from retrosplenial cortex ($U_{rn}(t)$ and $U_{ra}(t)$ respectively) do not contain the SD wave itself. This means that we analyzed not the signal properties at the wave propagation, but connectivity dynamics caused by wave propagation.

**Fig 1:**
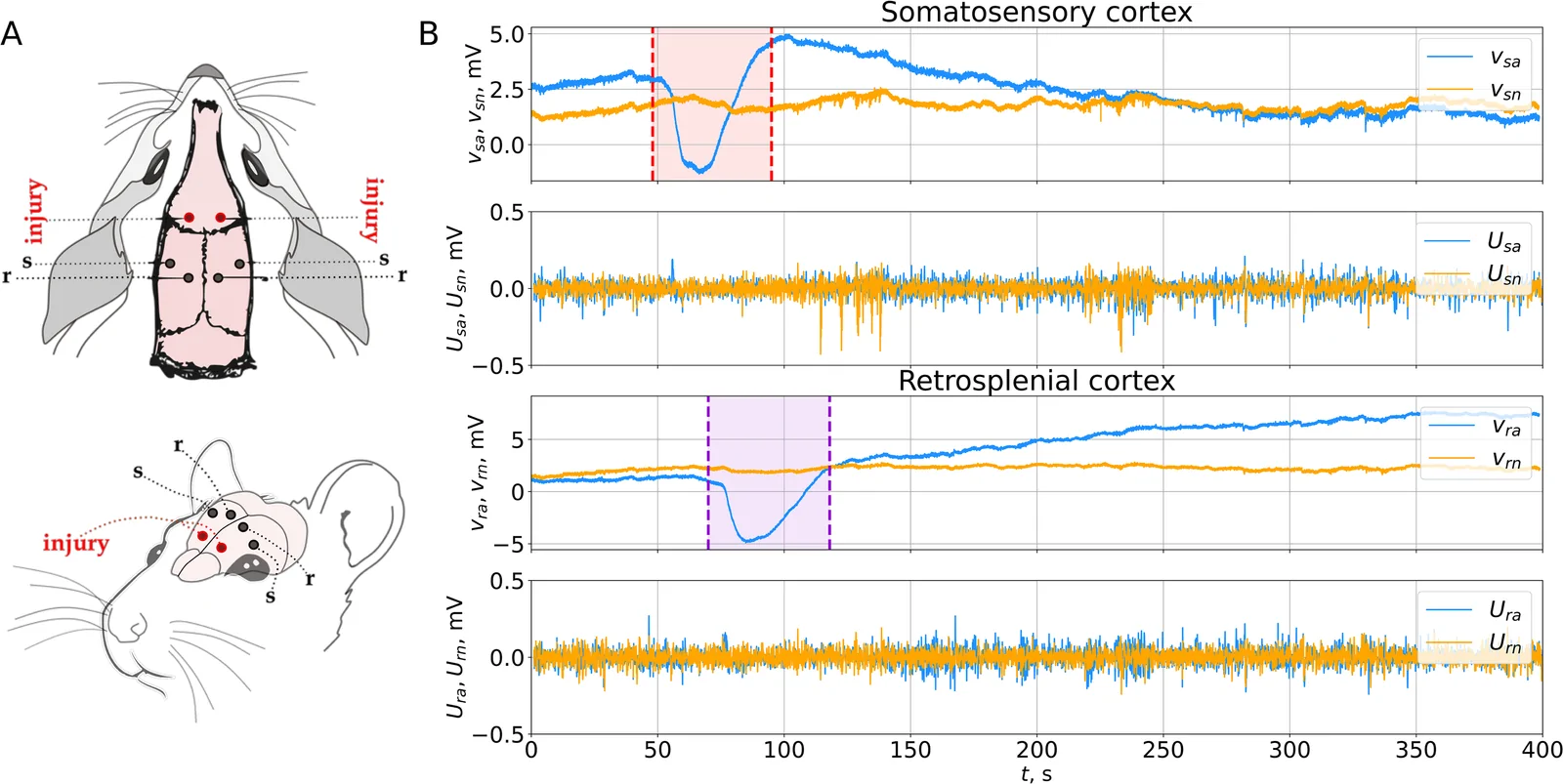
Typical AC/DC recordings of cortical activity after a focal cortical damage in an awake rat. A — location of focal injury of the frontal cortex (injury) and recording electrodes implanted in the somatosensory (*s*) and retrosplenial (*r*) cortex of the left and right hemispheres. B — time series of the original local field potential (LFP) signals *v*(*t*) and high-frequency components *U*(*t*) (detrended, f≥0.5 Hz, subplots 2 and 4) of LFP signals from homotopic areas of the somatosensory ($U_{s}$) and retrosplenial ($U_{r}$) cortex of the nonaffected ($U_{n}$) and affected ($U_{a}$) hemispheres after a microinjury of the left frontal cortex at time *t* = 0. Negative DC potential shift (SD) is marked by shaded area: red and violet for the somatosensory and retrosplenial cortices, respectively, on subplots 1 and 3.

### Mutual information function

Fig 2 demonstrates dynamics of mutual information (*MI*), the indirected measure of functional connectivity, for interhemispheric connections for the somatosensory and retrosplenial cortex.

**Fig 2:**
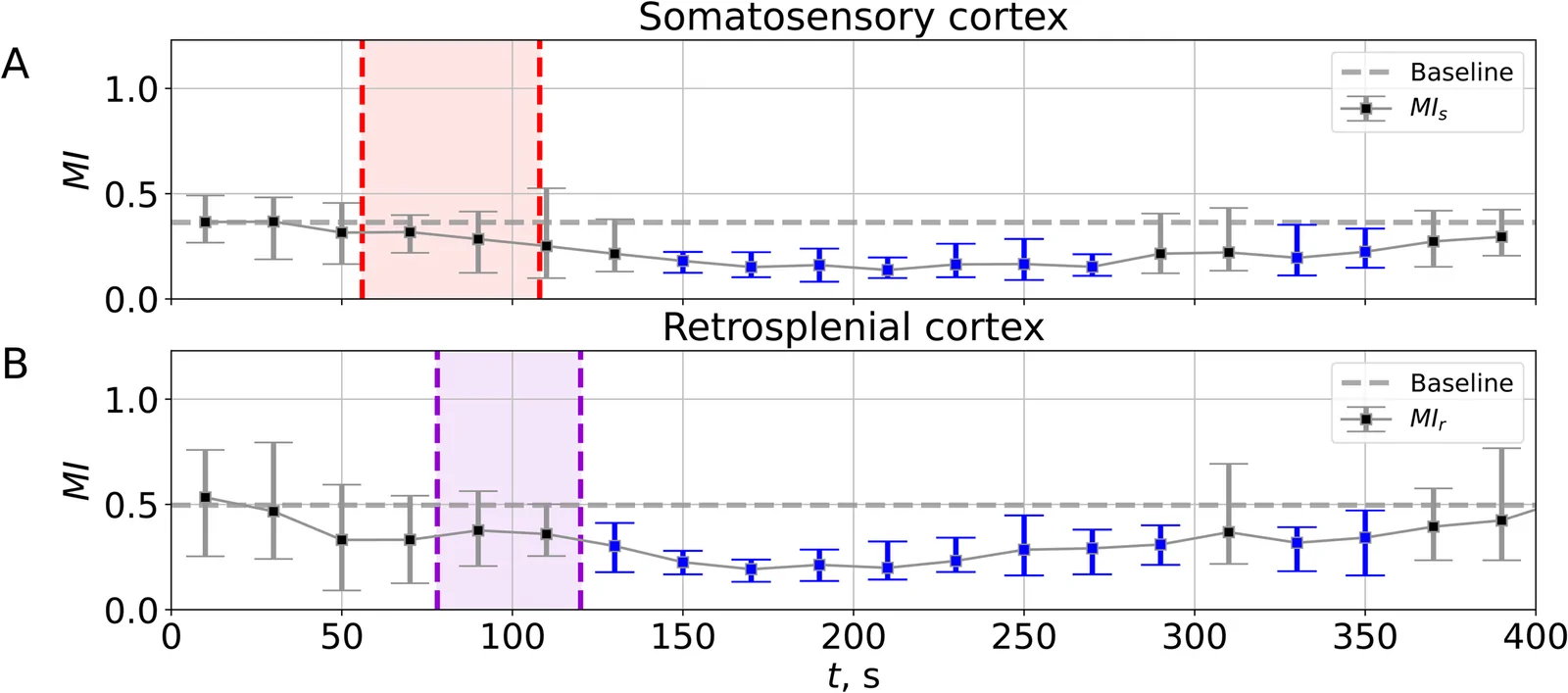
Dynamics of the mutual information function (*MI*) for symmetric interhemispheric connections, calculated in the nonoverlapping windows of *T* = 20 s length after focal injury of the frontal cortex (point *t* = 0). The injury triggered SD that propagated to intact somatosensory and retrosplenial regions of the cortex. The mean (per channel) duration of SD (DC shift) is marked by vertical shaded areas — red for the somatosensory cortex and violet for the retrosplenial one. The squares show the mean *MI* values averaged over *L* = 6 recordings. The errorbars mark minimal and maximal *MI* values. Horizontal dashed lines show the mean *MI* values calculated from the whole baseline activity of all rats and recordings for each channel pair separately. Significant (according to the written statistical criteria) results are colored in blue.

First, let us note that the mean *MI* value for the retrosplenial cortex is much (1.36 times) larger than for the somatosensory cortex: $MI_{s}=0.36389\pm 0.01019$, $MI_{r}=0.49639\pm 0.00843$ (mean ± std are given, *p*-value < 10^–24^). The mean value of *MI* between the hemispheres did not change during the SD depolarization phase (marked by shaded area), but started to reduce after SD termination.

After unilateral SD, the mean value of *MI* in somatosensoty cortex decreased during two long time intervals: for t∈[140;280] s (7 epochs of 20 s length in a row) and for t∈[320;360] s — two sequential 20 s epochs. Non-parametric min-max criterion showed that these results were very significant ($p=1.2\cdot 10^{-7}$). For the first (longest) decrease, the minimal value was ≈1.8 times lower than the mean baseline value and the standard deviation also dropped very much indicating that the values became very similar in different rats and recordings. This suggests that the drop is a time-locked event associated (at the population level) with much more independent activity of the hemispheres after injury associated with SD compared to the baseline state. In the retrosplenial cortex, we observed a prolonged decrease during nine sequential 20 s time epochs, t∈[120;300] s followed by a shorter (two epochs) decrease for t∈[320;360] s (synchronously with the late decrease in the somatosensory cortex). The maximum value of *MI* drop for the epoch t∈[160;180] was ≈2.1 times lower than the baseline. So, the drop in synchrony between the right and left retrosplenial cortical regions was even more pronounced in terms of both time and absolute value than in the somatosensory cortex.

The dynamics of *MI* for intrahemispheric connectivity significantly differed from that between the hemispheres. In the nonaffected hemisphere, *MI* level did not differ from the baseline during the considered time interval (Fig 3B). Affected cortex exhibited a sharp increase in *MI* when SD invaded both cortical regions — for t∈[80;120] s. It better matched the timing of SD arrival to the retrosplenial cortex than to the somatosensory cortex (Fig 3A). So, SD caused immediate hypersynchronization in the affected hemisphere that is followed by a decrease in similarity between hemispheres.

**Fig 3:**
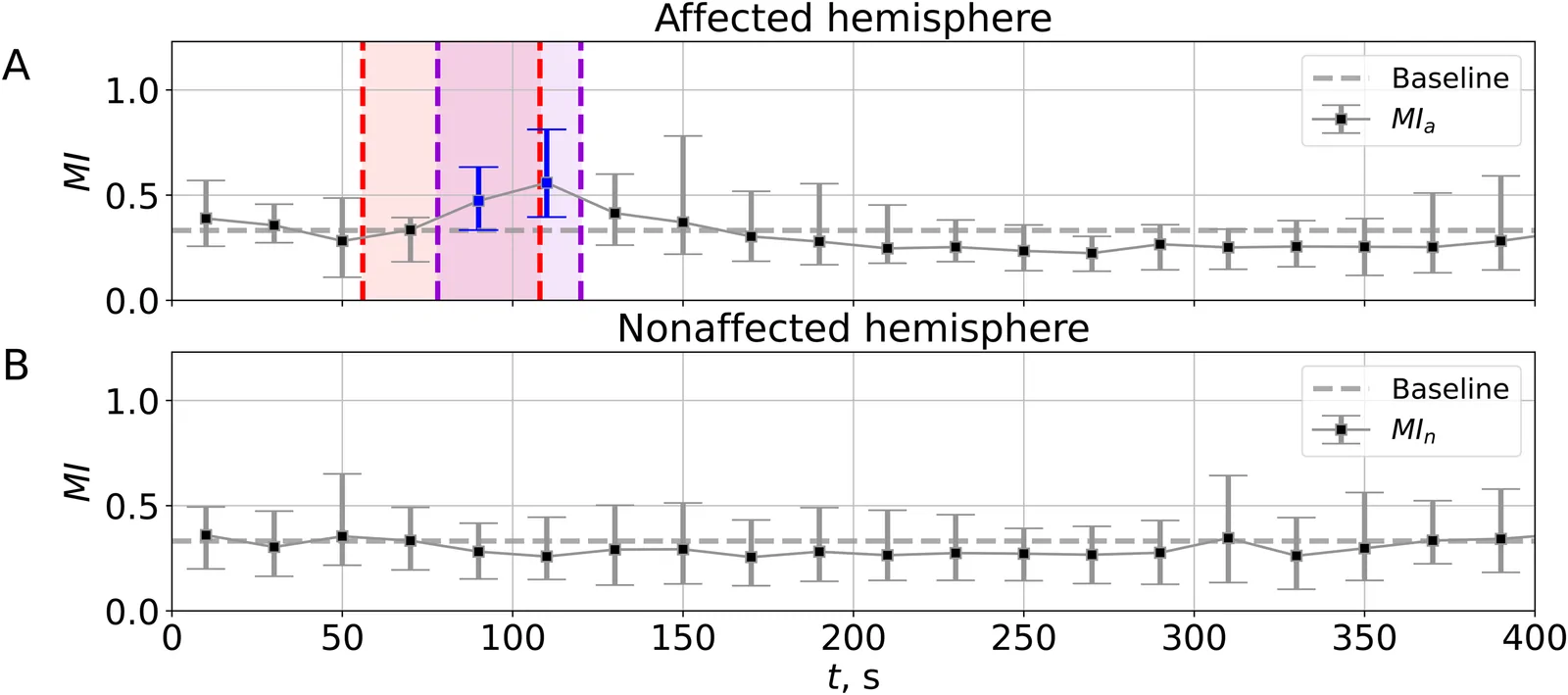
The dynamics of the mutual information function (*MI*) for intrahemispheric pairs, i.e., between the somatosensory and retrosplenial regions of the cortex, observed after frontal cortical injury triggering SD within affected (injured, A) and non-affected (uninjured, B) hemispheres. Vertical dashed lines mark the onset and termination of depolarization phase of SD in the somatosensory (red lines) and retrosplenial (violet lines) cortex in the affected hemisphere averaged over all recordings. Dashed horizontal line shows the mean value of *MI* for the baseline activity. Vertical errorbars indicate minimum and maximum of *MI* for each epoch, with its mean value being plotted by black squares. Errorbars corresponding to significant difference from the baseline are colored in blue.

### Transfer entropy

First, in the somatosensory cortex, the mean values of *TE* between the hemispheres during the baseline period were the same in both directions and remained unchanged till the end of SD (Fig 4). After the termination of SD, a significant unidirectional increase from the nonaffected hemisphere to the affected one appeared at two epochs: t∈[120;160] and t∈[180;220] (the break was a result of a single recording and possibly the increase was continuous) (Fig 4B). The increase in the opposite direction occurred to be insignificant.

**Fig 4:**
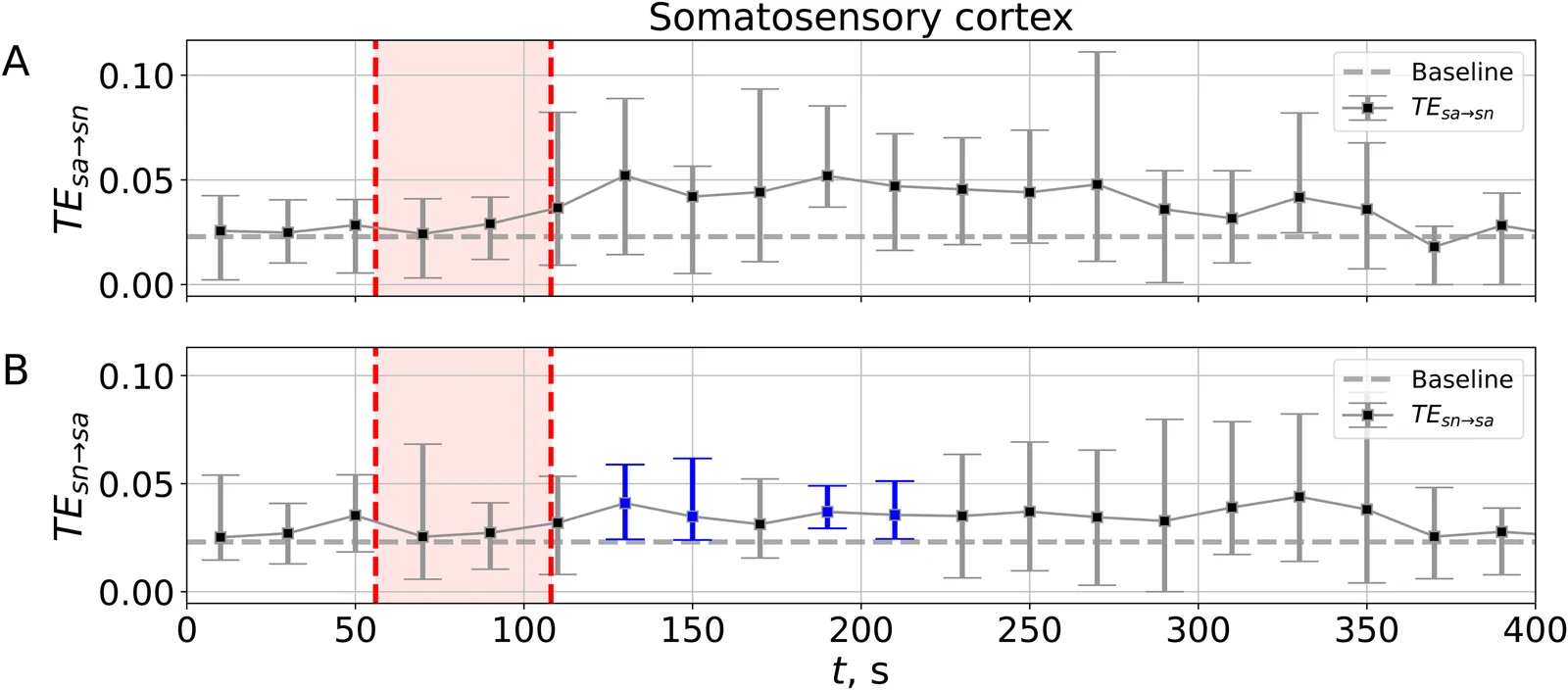
Effect of injury-induced SD on directional connectivity between the somatosensory cortical regions of the two hemispheres. Values of the transfer entropy (*TE*) were calculated in 20-s nonoverlapping time intervals for both directions: from the affected hemisphere to nonaffected one (A), and from the nonaffected hemisphere to the affected one (B). The average (black squares), minimal and maximal (by errobars) values are shown (*L* = 6). The horizontal dashed lines show the mean value calculated from the whole baseline activity of all rats. The mean duration of SD is marked by red vertical dashed lines.

An assessment of transfer entropy values for the retrosplenial cortex (Fig 5) revealed a bidirectional connectivity increase, with the baseline values being the same for both directions. The values of $TE_{ra\to rn}$ and $TE_{rn\to ra}$ did not differ before and during SD wave, but connectivity rose immediately after wave termination. In both directions the connectivity evolution was similar with local differences: first, longer increase (t∈[120;200] for *TE*_*ra*_→_*rn*_ and t∈[120;180] for *TE*_*rn*_→_*ra*_), than temporal return to baseline and a new rise for two epochs, see Fig 5 for details. So, the connectivity dynamics in retrosplenial cortex significantly differed from that in the somatosensory one, i.e., different cortical areas responded to SD wave differently.

**Fig 5:**
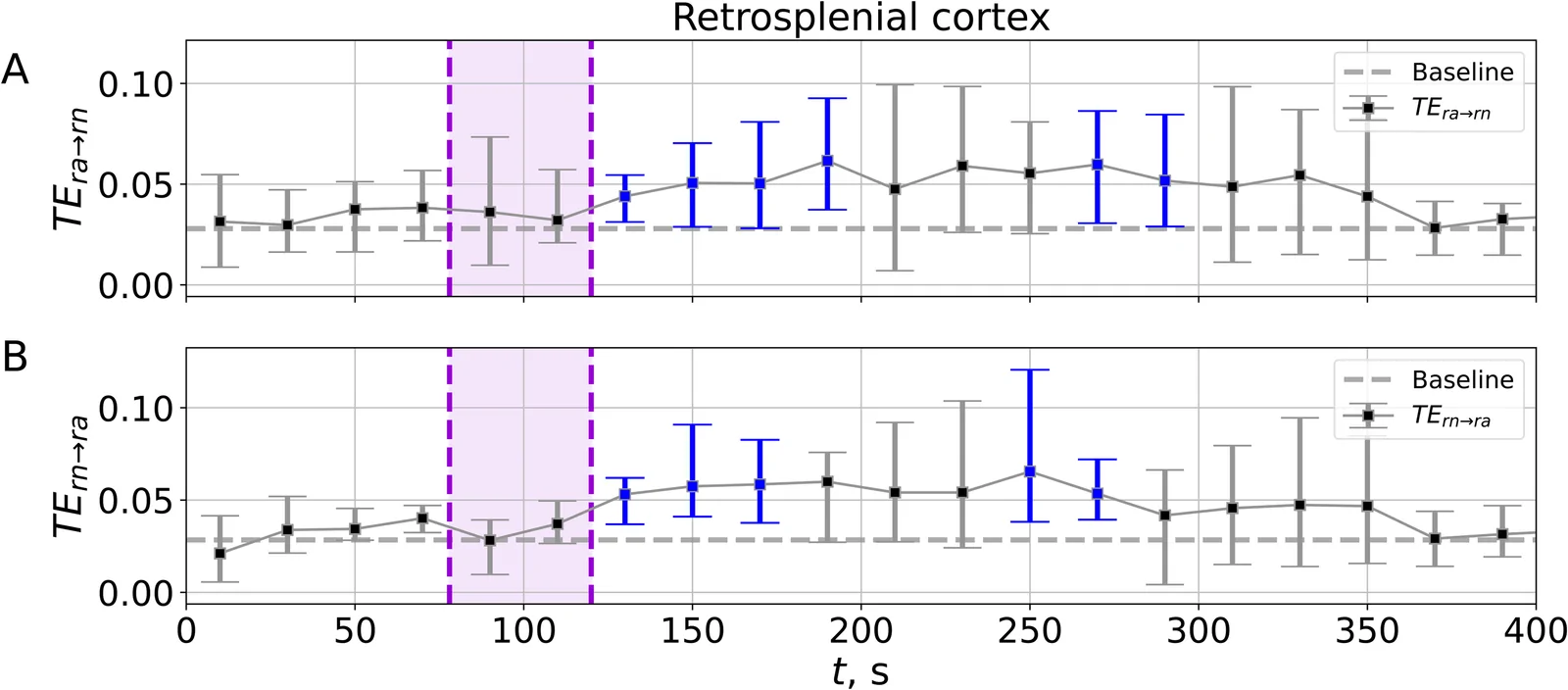
Effect of injury-induced SD on directional connectivity between retrosplenial cortical regions of the two hemispheres. Values of the transfer entropy (*TE*) were calculated in 20-s nonoverlapping time intervals for both directions: from the affected hemisphere to nonaffected one (A), and from the nonaffected hemisphere to the affected one (B). The average (black squares), minimal and maximal (by errobars) values are shown (*L* = 6). The horizontal dashed lines show the mean value calculated from the whole baseline activity of all rats. The mean duration of SD is marked by violent vertical dashed lines.

Next, let us consider intrahemispheric connectivity. In the affected hemisphere the baseline level of *TE* in both directions was the same and there were no significant changes until SD terminated. The only significant effect occurred 20 s after the termination of SD in the retrosplenial cortex (in the somatosensory one it ended even earlier): for three epochs (t∈[140;200]) one can see a unidirectional rise of the coupling — only for $TE_{sa\to ra}$ (Fig 6). All other increases were insignificant. This finding yet another time indicates the different role of cortical regions in processes of restoring to normal connectivity in the brain after SD wave.

**Fig 6:**
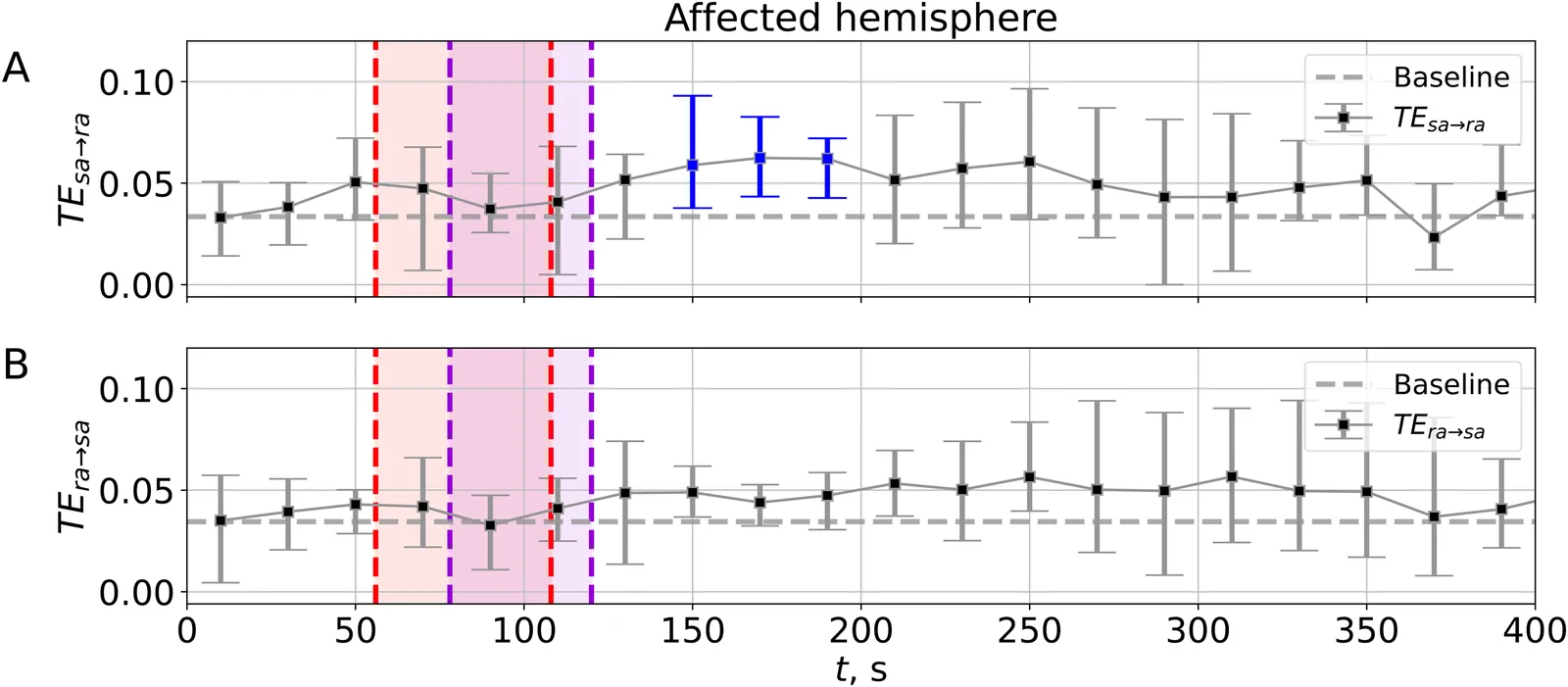
Effect of injury-induced SD on directional connectivity between somatosensory and retrosplenial cortical regions within the affected hemisphere. Values of the transfer entropy (*TE*) were calculated in 20-s nonoverlapping time intervals for both directions: from the somatosensory to retrosplenial cortex (A), and from the retrosplenial to the somatosensory cortex (B). The average (black squares), minimal and maximal (by errobars) values are shown (*L* = 6). The horizontal dashed lines show the mean values calculated from the whole baseline activity of all rats. The mean duration of SD is marked by vertical dashed lines.

In the nonaffected hemisphere, there was no significant connectivity change, see Fig 7 — both subplots. This well matches with the fact that the synchrony measured with *MI* also did not change throughout the whole considered time in the uninjured hemisphere (Fig 3B).

**Fig 7:**
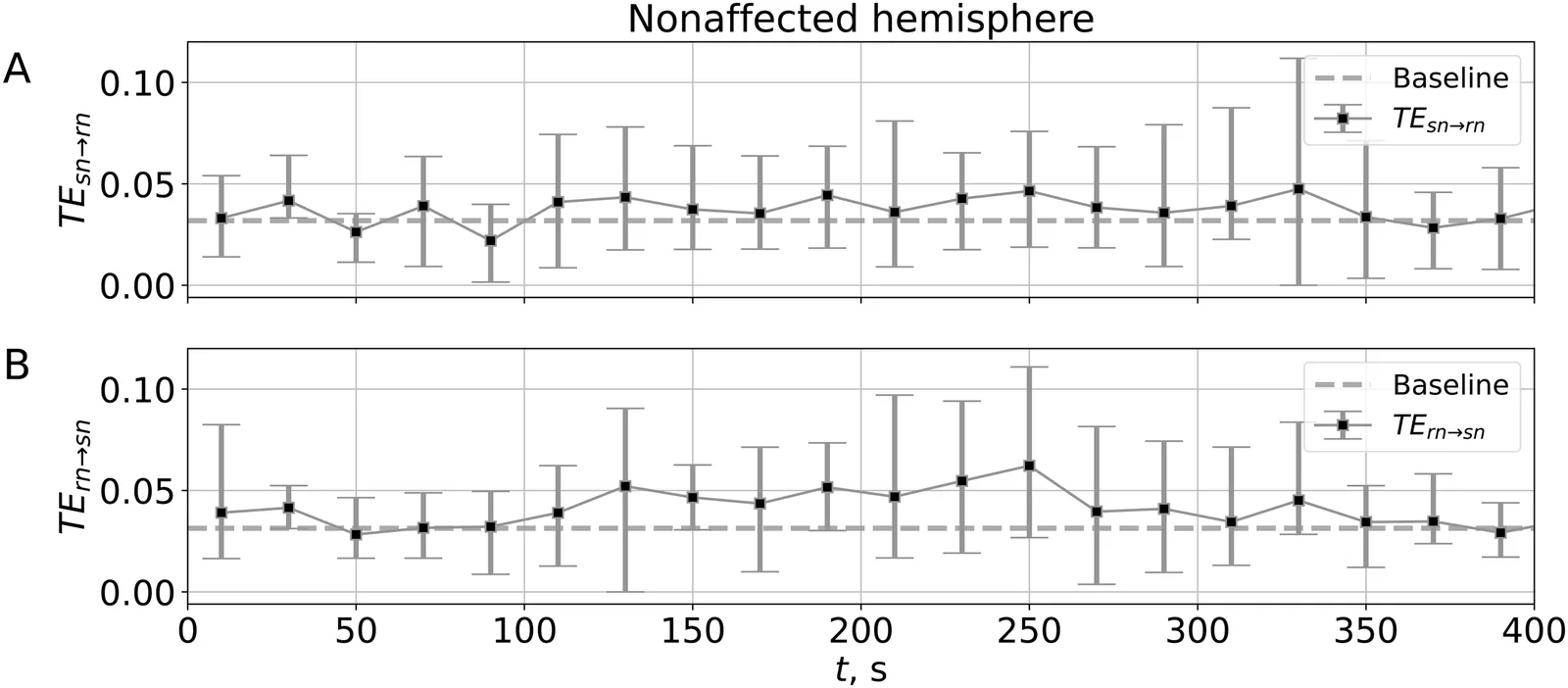
Connectivity dynamics in the nonaffected hemisphere during and after the SD wave propagation. Values of the transfer entropy (*TE*) were calculated in 20 s nonoverlapping time intervals in both directions: from the somatosensory to retrosplenial cortex (A), and from the retrosplenial to the somatosensory cortex (B). The average (black squares), minimal and maximal (by errobars) values are shown (*L* = 6). The horizontal dashed line shows the mean value calculated from the whole baseline activity of all rats. The mean duration of SD is marked by red vertical dashed lines.

## Discussion

The present study shows high functional plasticity of the cortical networks in the awake brain and their widespread reorganization in response to unilateral dysfunction of the cortex. We have found that focal cortical injury can produce distant connectivity changes via SD that is triggered by the injury, propagates to remote intact regions of the cortex and changes functional and effective connectivity in the regions.

Analysis of mutual information function has shown that injury-induced SD produces connectivity changes in distal cortical regions not directly affected by injury. The changes evolve over time from initial hyperconnectivity within the injured hemisphere (at the second minute post-injury) to interhemispheric hypoconnectivity (from the third through the sixth minute post-injury). No changes have been found in the contralesional cortex unaffected by SD. Transient interhemispheric decoupling after unilateral SD was also reported for the frontal [10], parietal [11] and occipital [12] cortical regions of awake rats. In anesthetized mice, optical imaging also showed reduced interhemispheric connectivity in the somatosensory and motor cortices after chemically-triggered SD [42]. Thus, the disruption of functional bihemispheric integrity is a reliable response of multiple cortical areas to SD. Interhemispheric interactions play a key role in ensuring cognitive functions and processing of sensory information. An effective interaction between the cortex of the two hemispheres is necessary to maintain a normal level of consciousness and attention. In patients with unilateral cortical stroke, the functional connectivity between the homotopic areas of the two hemispheres is acutely disrupted and the disruption correlates with the attention deficit in a visual task [43].

Analysis of effective connectivity (transfer entropy) has revealed that during the post-SD decoupling detected by mutual information function, influence from the uninjured cortex to the injured one increases in both somatosensory and retrosplenial cortices. Previously, we have described similar connectivity changes in the frontal cortex and the key role of cortical inhibition in the SD-induced network alterations [10]. We found that pharmacological weakening of inhibitory transmission attenuated or completely blocked the changes in interhemispheric connectivity produced by SD. The enhanced drive from the uninjured cortex could contribute to recovery/compensation of unilateral cortical dysfunction produced by SD. We can speculate that the described alterations of intracortical functional interactions reflect homeostatic network plasticity and be involved in connectional diaschisis after acute brain injury.

The summary of all SD-induced changes in undirected (similarity/synchrony) and directed (causality) connectivity described in the present study are schematically plotted in Fig 8. The long-lasting decrease in interhemispheric synchrony (*MI*) is accompanied by increased causality that can be explained in terms of brain plasticity. The sites of the unaffected (contralesional) cortex are working to compensate the synchrony drop caused by SD wave and to normalize brain activity after transient dysfunction induced by SD. At the same time, bidirectional increase in interhemispheric coupling detected in the retrosplenial cortex counteracts the synchrony drop for a longer post-SD period (possibly another mechanism).

**Fig 8:**
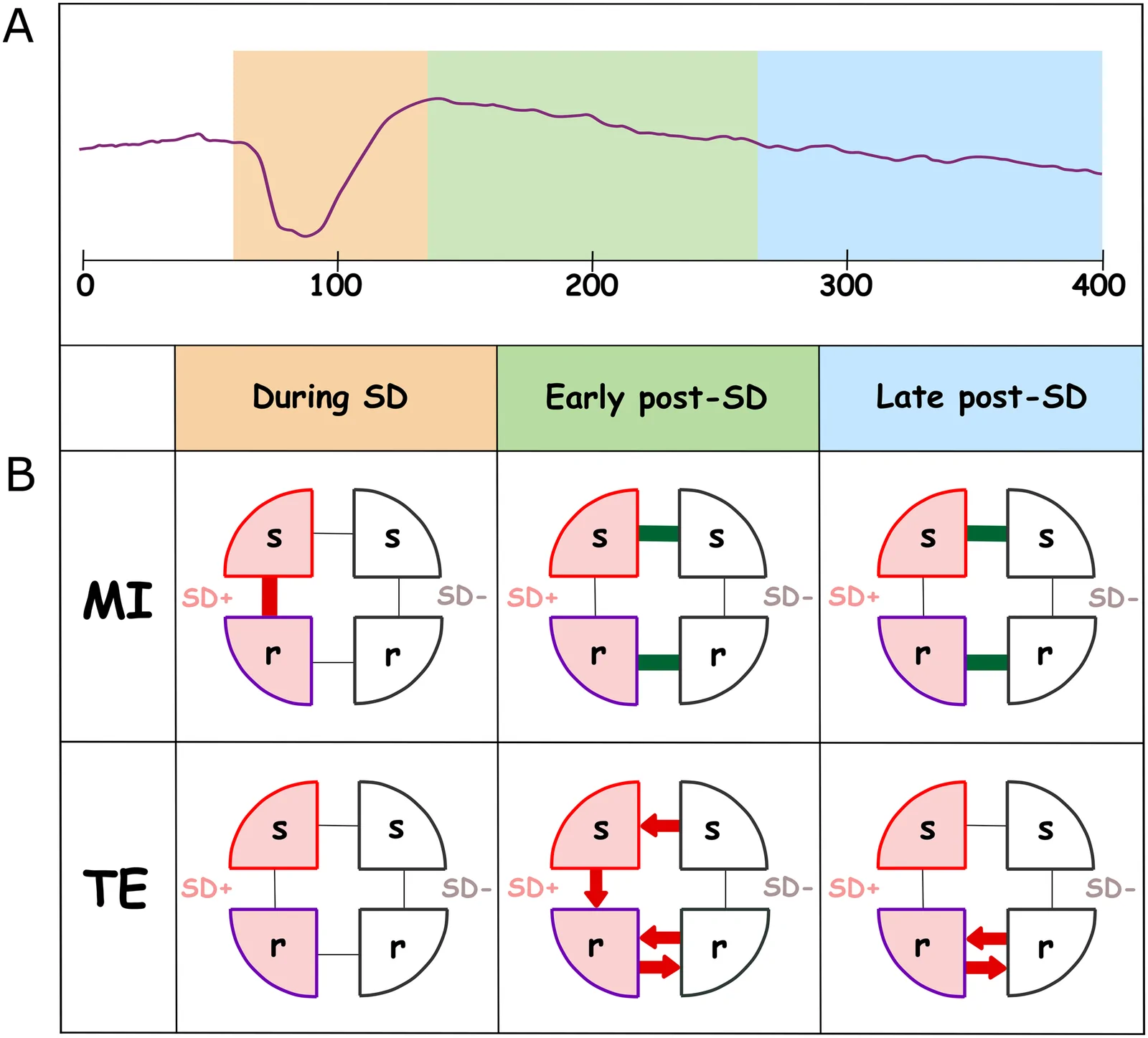
Evolution of connectivity and similarity (synchrony) induced by unilateral cortical spreading depolarization wave. A – time line of cortical SD that marks three period with significant connectivity changes (during SD, early post-SD and late post-SD). B – changes in connectivity detected by two measures (*MI* and *TE*). Red lines/arrows indicate an increase in connectivity between somatosensory (s) and retrosplenial (r) cortex of the hemispheres affected (SD+) and unaffected (SD-) by SD. Green lines indicate a decrease in connectivity/similarity between the regions.

Functional connectivity in the retrosplenial cortex and its alterations produced by SD differ from those detected in the somatosensory cortex. First, analysis of mutual information function has demonstrated that the retrosplenial cortex exhibits significantly stronger baseline interhemispheric connectivity than other cortical regions — the somatosensory cortex in the present study and the motor cortex in [10]. The strong baseline functional connectivity between homotopic sites of the retrosplenial cortex corresponds well to its known role as a component of the default-mode network (DMN) — network consisting of highly interconnected nodes at rest. Next, we show that cortical SD strongly disturbs connectivity balance in the retrosplenial cortex. In the region, SD-induced interhemispheric decoupling lasts longer and is accompanied by enhanced influence from the injured cortex to the intact one that has never been observed in other cortical regions [10]. In humans, reduced fMRI connectivity within the DMN, particularly in the posterior cingulate cortex (analog of the retrosplenial cortex in rodents), was reported shortly after mild traumatic brain injury and the reduced posterior connectivity correlated with neurocognitive dysfunction [44]. Given clinical evidence for early cognitive deficit and regular occurrence of cortical SD after acute brain injury [45], the effects of injury-induced SD on DMN connectivity found in the current study could contribute to cognitive dysfunction after brain insults. The results of the study provide new insights into pathophysiological mechanisms of acute brain injury, its clinical symptoms and recovery processes. The data may be used for identification of novel therapeutical targets and development of new treatment strategies.
